# Converging TLR9 and PI3Kgamma signaling induces sterile inflammation and organ damage

**DOI:** 10.1038/s41598-019-55504-0

**Published:** 2019-12-13

**Authors:** Braulio Henrique Freire Lima, Pedro Elias Marques, Lindisley Ferreira Gomides, Matheus Silvério Mattos, Lucas Kraemer, Celso M. Queiroz-Junior, Mark Lennon, Emilio Hirsch, Remo Castro Russo, Gustavo Batista Menezes, Edith M. Hessel, Augustin Amour, Mauro Martins Teixeira

**Affiliations:** 1 Department of Biochemistry and Immunology, Institute of Biological Sciences, Feredal University of Minas Gerais, Belo Horizonte, Minas Gerais Brazil; 2 Center for Gastrointestinal Biology, Instituto de Ciências Biológicas, Feredal University of Minas Gerais, Belo Horizonte, Minas Gerais Brazil; 3 Physiology and Biophysics/Instituto de Ciencias Biologicas, Feredal University of Minas Gerais, Belo Horizonte, Minas Gerais Brazil; 4 Departament of Morphology, Institute of Biological Sciences, Feredal University of Minas Gerais, Belo Horizonte, Minas Gerais Brazil; 5 Target Sciences, GlaxoSmithKline, Stevenage, Hertfordshire, Stevenage, United Kingdom; 6 Department ot Molecular Biotechnology and Health Sciences, Molecular Biotechnology Center, University of Torino, Torino, Italy; 7 Refractory Respiratory Inflammation DPU, GlaxoSmithKline, Hertfordshire, Stevenage, United Kingdom

**Keywords:** Phosphoinositol signalling, Toll-like receptors

## Abstract

Toll-like receptor 9 (TLR9) and Phosphatidylinositol-3-kinase gamma (PI3Kγ) are very important effectors of the immune response, however, the importance of such crosstalk for disease development is still a matter of discussion. Here we show that PI3Kγ is required for immune responses in which TLR9 is a relevant trigger. We demonstrate the requirement of PI3Kγ for TLR9-induced inflammation in a model of CpG-induced pleurisy. Such requirement was further observed in inflammatory models where DNA sensing via TLR9 contributes to disease, such as silicosis and drug-induced liver injury. Using adoptive transfer, we demonstrate that PI3Kγ is important not only in leukocytes but also in parenchymal cells for the progression of inflammation. We demonstrate this crosstalk between TLR9 and PI3Kγ *in vitro* using human PBMCs. The inhibition of PI3Kγ in CpG-stimulated PBMCs resulted in reduction of both cytokine production and phosphorylated Akt. Therefore, drugs that target PI3Kγ have the potential to treat diseases mediated by excessive TLR9 signalling.

## Introduction

Toll-Like receptors (TLRs) are type I conserved transmembrane proteins containing a leucine-rich region and an intracellular domain resembling the IL-1 receptor (IL-1R)^1,2^. These receptors recognize a wide variety of highly conserved structural molecules, namely pathogen associated molecular patterns (PAMPS) and host molecules linked to injury or damage associated molecular patterns (DAMPS). The detection of PAMPs or DAMPs initiates a host immune response against the insult, which constitutes one of the fundamental mechanisms of innate immunity^3–5^. The 13 and 10 TLRs reported respectively in mice and humans can be divided into three groups according to their cellular location: at the plasma membrane (TLRs 1; 2; 5; 6; 10); in endosomes (TLRs 3; 7; 8; 9; 11; 12; 13); or a third group consisting only of TLR4, which is found both at the plasma membrane or in intracellular vesicles^6^.

Upon binding of PAMPs/DAMPs, TLRs dimerize and its intracellular domain is phosphorylated notably on Tyrosine residues by a variety of kinases including SRC family kinases^7^. Subsequently, adaptor molecules such as MAL/MyD88 or TRIF couple to the receptors and start a cascade of additional phosphorylation and ubiquitination that culminate with the translocation of NF-κB or IRFs into the nucleus where they promote the transcription of pro-inflammatory cytokines (IL-1β, TNF-α, IL-6 IFN-γ and others) and type I interferons (IFNs)^4,5^. Although TLRs are very important molecules for the initiation of the immune response by the innate system, their over activation can be detrimental to the host^8^. Hence, there are several reports to suggest that hyper responsiveness of TLRs is associated with disease severity in the case of sepsis, liver damage, psoriasis, inflammatory bowel diseases, multiple sclerosis, lupus, arthritis and Alzheimer’s disease^4,9–13^.

Phosphatidylinositol-4,5-bisphosphate 3-kinases (PI3Ks) are a family of intracellular lipid kinases whose function is to catalyse the phosphorylation of the OH group located in the position 3 of the phosphatidylinositol ring^14^. To date, there are 8 known PI3K isoforms in mammals and they are divided into three different classes based on structure similarities. Class I PI3K, the most studied class of PI3K, consists of a heterodimer composed of a catalytic domain called p110 and one of the various existing regulatory subunits. Class I PI3Ks are further divided into two subclasses: Class IA composed of PI3Kα, PI3Kβ and PI3Kδ and Class IB for which PI3Kγ is the unique member. Class IA PI3Kα, PI3Kβ and PI3Kδ are respectively formed by the interaction of their catalytic subunits p110α, β or δ with one of the regulatory subunits p85α, p85β, p55, p55β or p55γ. In parallel, Class IB PI3Kγ is composed by its catalytic subunit p110γ and one of its regulatory subunits p101 or p87. Although PI3Kα and PI3Kβ are constitutively expressed in all tissues, expression of PI3Kδ is more restricted to hematopoietic cells and PI3Kγ expression is almost exclusive to leukocytes^15^.

The PI3K pathway is involved in a wide variety of cellular function such as protein synthesis (via mTORC1), survival (via FoxO1 and Bax), cell cycle (via p21 and p27), cell metabolism (via glycogen synthase), cell migration, T and B receptor signalling and G-coupled protein receptors activation, such as chemokine receptors^15–22^. Accordingly, knockout p110α or β mice are embryonic lethal whereas mice deficient for p110γ or δ reach adulthood with no altered phenotype except for impaired stimulation of their immune system^23–26^.

The PI3K pathway has been shown to be activated by various TLR ligands such as CpG, LPS, flagellin and by-products of viral infection^27–30^, however, its modulatory role in TLR activation has been shown to be either positive or negative depending on the circumstances^31,32^. Previous work by Guidicci and collaborators showed that PI3Kδ inhibition dampened the TLR9-induced production of type I IFN but not of pro-inflammatory cytokines by plasmacytoid pre-dendritic cells^33^. In addition, other authors have also reported that inhibition of PI3Ks resulted in limiting the immune response triggered by TLRs^33–36^. In contrast, conflicting reports have suggested that PI3Ks act as a suppressor of TLR signalling and that, upon inhibition of the PI3K enzyme, the TLR response is enhanced^37–40^. Here we turned our attention to PI3Kγ and examined its function in mediating TLR9-induced inflammatory responses both *in vivo* in mouse models or *in vitro* in peripheral blood mononuclear cells (PBMCs). We observed that PI3Kγ has a critical role in TLR9-mediated inflammatory cell recruitment, disease progression and cytokine production. We demonstrate that targeting TLR9 or PI3Kγ appear as promising approaches to control sterile organ damage provoked by toxic irritants such as silica-induced lung fibrosis and drug-induced liver injury since these were dependent on TLR9 receptor and PI3Kγ activity.

## Results

### CpG injection in the pleural cavity induces cell recruitment that is reduced in PI3Kγ^−/−^ and AS605240-treated mice

**Figure 1:**
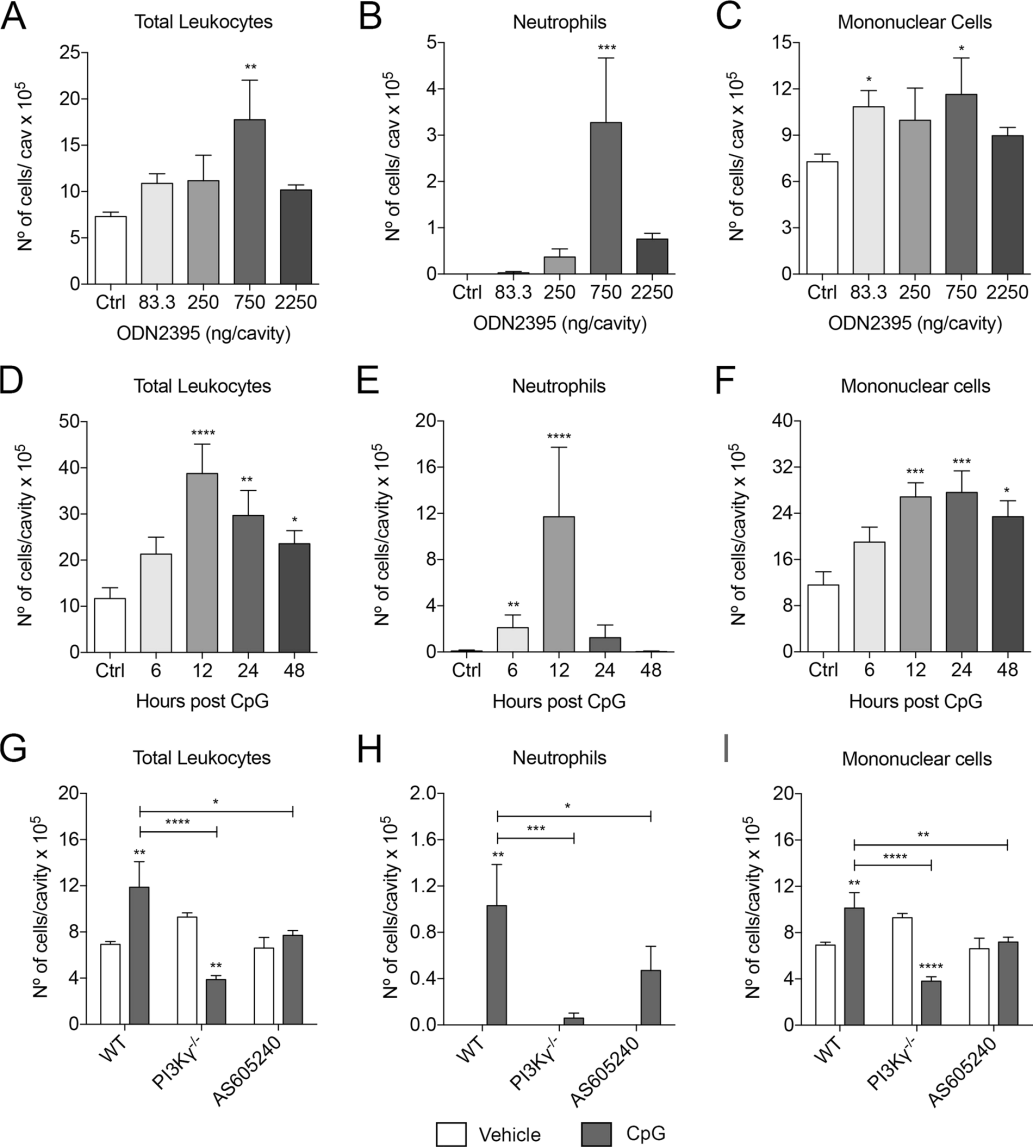
CpG induce PI3Kγ-dependent leukocyte recruitment. (**A–C**) The cellular infiltration evoked by CpG in a dose response manner: total cells (**A**), neutrophils (**B**) and mononuclear cells (**C**). Data represented as mean ± SEM. p-value was calculated using one-way ANOVA test with Dunn’s uncorrected test; *p < 0.05; **p < 0.01 (n = 5 mice). (**D–F**) Time course recruitment of total cells (**D**), neutrophils (**E**) and mononuclear cells (**F**) induced by CpG (750 ng/cavity). Data represented as mean ± SEM. p-value was calculated using one-way ANOVA test with Dunn’s uncorrected test; *p < 0.05; **p < 0.01; ***p < 0.001 (n = 7 mice). (**G–I**) Effect of PI3Kγ inhibition (AS605240 20 mg/Kg or KO) in the recruitment of total cells (**G**), neutrophils (**H**) and mononuclear cells (**I**) induced by CPG (750 ng/cavity). Data represented as mean ± SEM. p-value was calculated using two-way ANOVA test with uncorrected Fisher’s LSD test; *p < 0.05; **p < 0.01; ***p < 0.001; ****p < 0.0001 (n = 6 mice).

To determine whether TLR9 may cross-talk with PI3Kγ, we established a model of CpG induced pleurisy in mice. The pleural cavity is both free of microorganisms and has low number of resident immune cells. Thus, subtle changes in the cell composition induced by CpG could be easily noticeable. CpG treatment induced pleural inflammation after 24 hours in a dose dependent manner with an optimal dose at 750 ng/cavity (Fig. 1A). Moreover, the inflammation caused by CpG instillation was composed primarily of neutrophils and mononuclear cells (Fig. 1B,C). We then addressed the dynamics of the inflammation caused by CpG. Hence, 750 ng/cavity was instilled in the pleural cavity of the mice and the cellular recruitment was assessed over time. CpG-induced leukocyte recruitment started at 6 hours, peaked at 12 hours and began to decay at 48 hours (Fig. 1D). The analysis of neutrophils and monocytes during this process indicated that these two cell types behaved differently. Neutrophils migrated to the tissue as early as 6 hours and reached a peak of infiltration at 12 hours post CpG stimulation. At a 24-hour time point, neutrophil numbers were back down and equivalent to those at 6 hours. At 48 hours after CpG injection, neutrophils were no longer detected in the pleural cavity (Fig. 1E). On the other hand, the kinetics of monocytes in this model were rather different. As for neutrophils, these cells were detectable in the pleural cavity at 6 hours post stimulus and numbers peaked at 12 hours post CpG stimulation. However, the mononuclear cell numbers in the cavity were maintained until 24 hours after the insult and started to decay only at 48 hours after the stimulation (Fig. 1F).

Finally, we investigated the role of PI3Kγ in the CpG-mediated pleurisy. WT, PI3Kγ^−/−^ or WT mice treated with AS605240 (a selective PI3Kγ inhibitor) animals were injected with 750 ng/cavity of CpG in the pleura and the composition of the leukocytes recruited to the cavity were analysed 12 hours after the injection. In WT mice, CpG induced inflammation as previously observed with cellular infiltration of neutrophils and mononuclear cells. However, PI3Kγ^−/−^ and AS605240-treated mice had less leukocytes recruited into the pleural cavity following CpG injection than equivalent untreated WT mice (Fig. 1G–I). For the mice treated with AS605240, the difference in total leukocytes appeared to be mostly driven by the reduced neutrophil recruitment since the migration of mononuclear cells had a lower effect by the inhibitor treatment in contrast to the PI3Kγ^−/−^ mice, for which both the neutrophils and mononuclear cells were reduced compared to the WT control mice.

### TLR9 deficiency significantly reduces lung inflammation and fibrosis induced by silica

**Figure 2:**
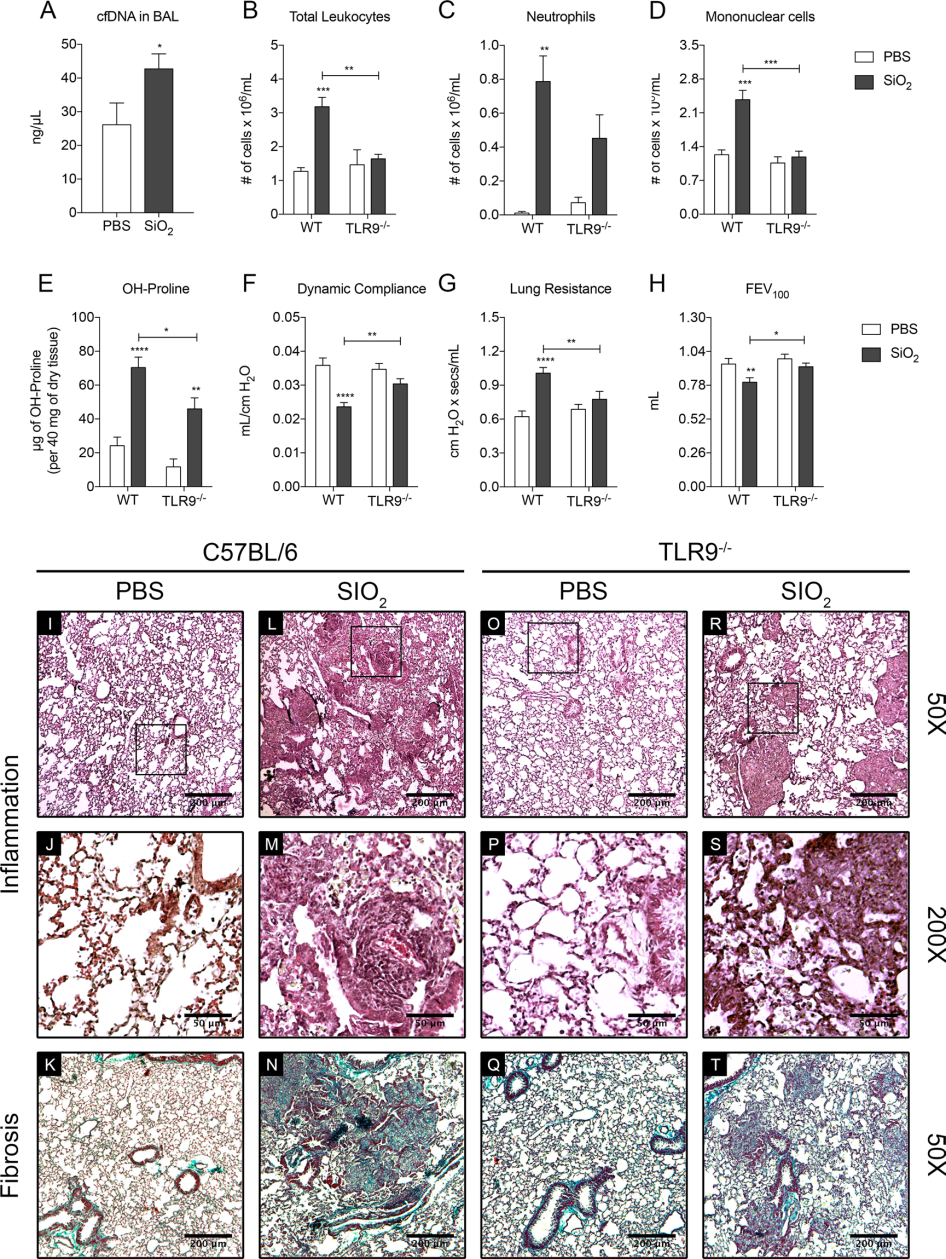
TLR9 deletion prevents silica inflammation and fibrosis. (**A**) Cell free DNA in PBS (n = 4) and in SiO2 (n = 5) exposed WT mice (10 mg). Data represented as mean ± SEM. p-value was calculated using one-tailed Student’s test; *p < 0.05. (**B**–**D**) Number of cells recovered in the BALF of WT (n = 6) and TLR9^−/−^ (n = 4) mice after silica exposure: total cells (**B**), neutrophils (**C**) and mononuclear cells (**D**). (**E**) Hydroxyproline content in the lung parenchyma of WT (n = 5) and TLR9^−/−^ (n = 4) mice. (**F**–**H**) Lung function parameters of WT (n = 12) and TLR9^−/−^ (n = 10) mice: Dynamic compliance (**F**), Lung Resistance (**G**) and Forced Expiratory volume at 100 ms (**H**). (**I** to **J**) Representative sections of lung lobes stained with Haematoxylin and Eosin (Inflammation) and Gomori’s Trichrome (Fibrosis). Bars, 200 µm for 50x magnification and 50 µm for 200×. Data represented as mean ± SEM. p-values were calculated using two-way ANOVA test with Dunn’s uncorrected test; *p < 0.05; **p < 0.01; ***p < 0.001.

The pleurisy model confirmed to us the role of PI3Kγ in mediating CpG-induced inflammation. We then checked the role of TLR9 in a model of airway silicosis prior to testing the PI3Kγ dependency of that model. After 28 days of silica exposure, a significant increase in the airway levels of cell free DNA (cfDNA) was observed in WT mice (Fig. 2A). We also observed a significant increase in the number of leukocytes infiltrated in the airways, which were composed of a mixture of both neutrophils and mononuclear cells (Fig. 2B–D). This was followed by fibrosis in the lungs of these mice as indicated by the increased levels of hydroxyproline (Fig. 2E). This silica-induced changes in the lungs resulted in organ dysfunction as shown by reduced compliance, increased resistance and lower FEV_100_ (Fig. 2F–H). In contrast, we found that silica exposure in TLR9^−/−^ mice resulted in comparatively less lung inflammation than WT mice. TLR9^−/−^ mice had fewer inflammatory cells in the BALF due to lower number of mononuclear cells (Fig. 2B–D). In addition, these animals had less lung fibrosis than WT mice, as indicated by hydroxyproline content in the lungs (Fig. 2E). We also observed that the combination of less lung inflammation and fibrosis resulted in better lung function. The TLR9 deficiency prevented the impairment of the lung mechanics caused by silica exposure in WT mice. For instance, TLR9^−/−^ mice had improved compliance, resistance and FEV_100_ measurements, which were more akin to the control group (Fig. 2F–H).

Tissue sections from lungs of WT mice showed that silica exposure induced diffuse inflammatory infiltrate and development of granulomas in several areas. This exposure also resulted in interstitial oedema and hyperplasia, leading to alveolar tissue thickening. Gomori Trichrome staining of tissue sections showed that collagen deposition in the lungs of WT mice was diffuse and co-localised with areas of inflamed tissue (Fig. 2I–N). In contrast, TLR9^−/−^ mice showed comparatively reduced lung inflammation and fibrosis. The lung parenchyma of these mice had less tissue thickening and, although present, smaller granuloma formation. Like WT mice, the low collagen staining in the lungs of TLR9^−/−^ mice co-localised with areas where inflammation was present (Fig. 2O–T).

### PI3Kγ^−/−^ mice have less silica-induced lung inflammation and fibrosis

**Figure 3:**
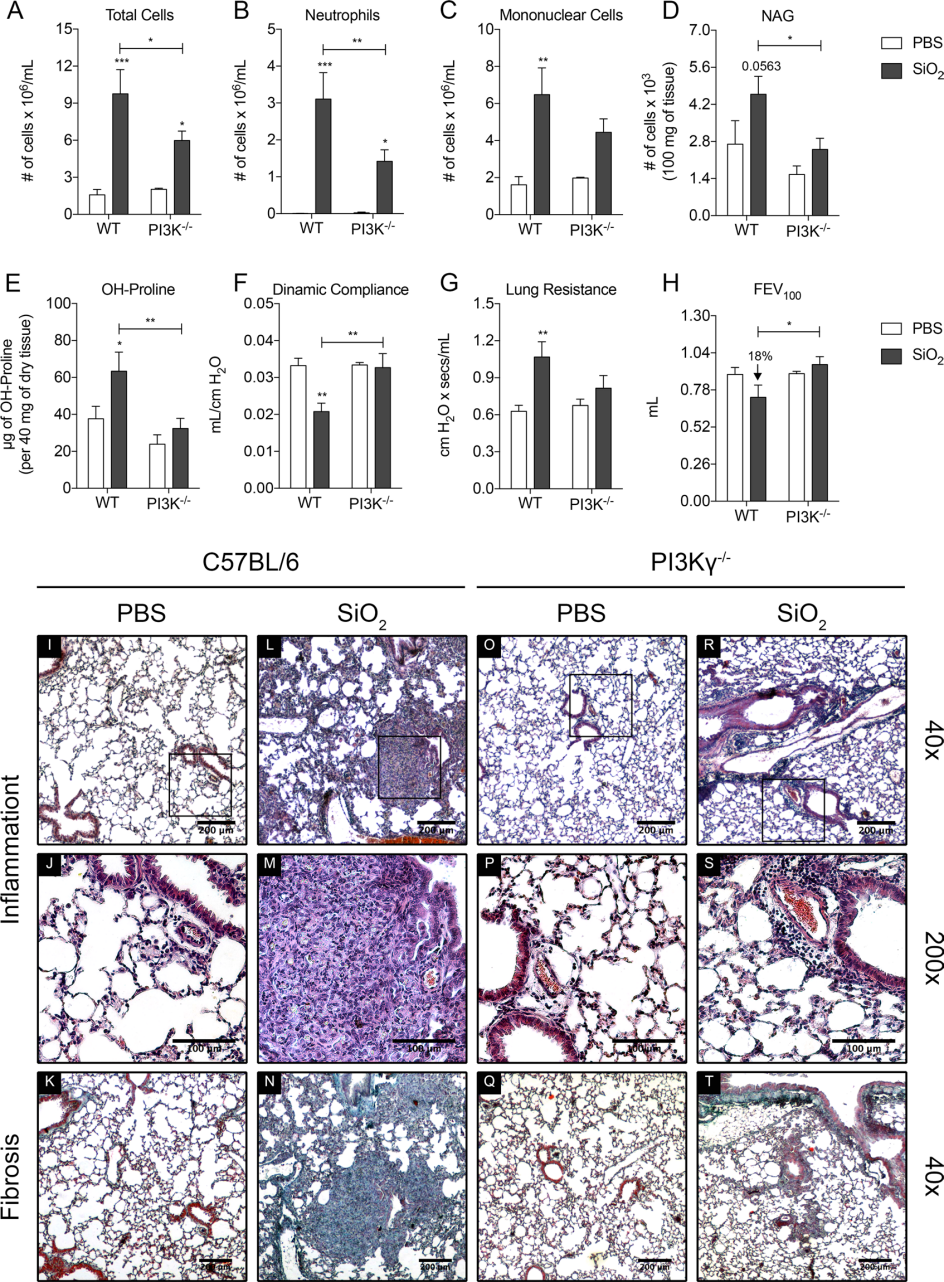
PI3Kγ deletion prevents silica inflammation and fibrosis. (**A**–**C**) Number of cells recovered in the BALF of WT (n = 5) and PI3Kγ^−/−^ (n = 6) mice after silica exposure (10 mg): total cells (**A**), neutrophils (**B**) and mononuclear cells (**C**). (**D**) NAG activity in the lung parenchyma. (**E**) Hydroxyproline content in the lung parenchyma of WT (n = 5) and PI3Kγ^−/−^ (n = 6) mice. (**F**–**H**) Lung function parameters of WT (n = 5) and PI3Kγ^−/−^ (n = 6) mice: Dynamic compliance (**F)**, Lung Resistance (**G**) and Forced Expiratory volume at 100 ms (**H**). (**I** to **J**) Representative sections of lung lobes stained with Haematoxylin and Eosin (Inflammation) and Gomori’s Trichrome (Fibrosis). Bars, 200 µm for 40x magnification and 100 µm for 200x. Data represented as mean ± SEM. p-value was calculated using two-way ANOVA test with uncorrected Fisher’s LSD test; *p < 0.05; **p < 0.01; ***p < 0.001.

To test the PI3Kγ dependency of the silicosis model, mice were exposed to 28 days of silica. WT mice showed leukocyte recruitment to the airways that was composed by a mixture of both neutrophils and mononuclear cells (Fig. 3A–C). These mice also showed both increased number of macrophages in the lung parenchyma and lung fibrosis as measured by the NAG activity and hydroxyproline assay, respectively (Fig. 3D,E). The combination of airway and tissue inflammation plus collagen deposition in the lungs resulted in worsening of lung function as shown by low lung compliance, high lung resistance and reduced FEV_100_ (Fig. 3F–H).

PI3Kγ^−/−^ mice showed reduced number of cells in the BALF due to fewer neutrophils and mononuclear cells (Fig. 3A–C). Moreover, they had lower accumulation of mononuclear cells and collagen deposition in the lung parenchyma (Fig. 3D,E). The reduced inflammation and lung fibrosis resulting from the absence of PI3Kγ rescued the lung dysfunction of these mice, as indicated by the normalised parameters of lung compliance, lung resistance and FEV_100_ (Fig. 3F–H).

As mentioned before, silica exposure causes lung inflammation, granuloma formation and fibrosis in WT mice (Fig. 3I–N). In contrast, PI3Kγ^−/−^ mice showed comparatively reduced lung inflammation. The lung parenchyma of these mice had less oedema and leukocyte accumulation was restricted to perivascular areas. These mice also had less staining for collagen, which was detected in the perivascular area, where inflammation was located (Fig. 3O–T).

### PI3Kγ^−/−^ and AS605240-treated mice are protected from drug-induced liver damage

We then used the established TLR9-dependent model of acetaminophen (APAP)-induced liver injury (DILI) to further evaluate the potential function of PI3Kγ in TLR9 activated tissue injury. We previously showed that, whilst the injury was initiated by APAP, the lesions were further amplified following the recruitment of neutrophils to the liver necrotic areas^10,41^. These observations led us to hypothesis that PI3Kγ inhibition would have a protective effect in the context of APAP-induced DILI.

**Figure 4:**
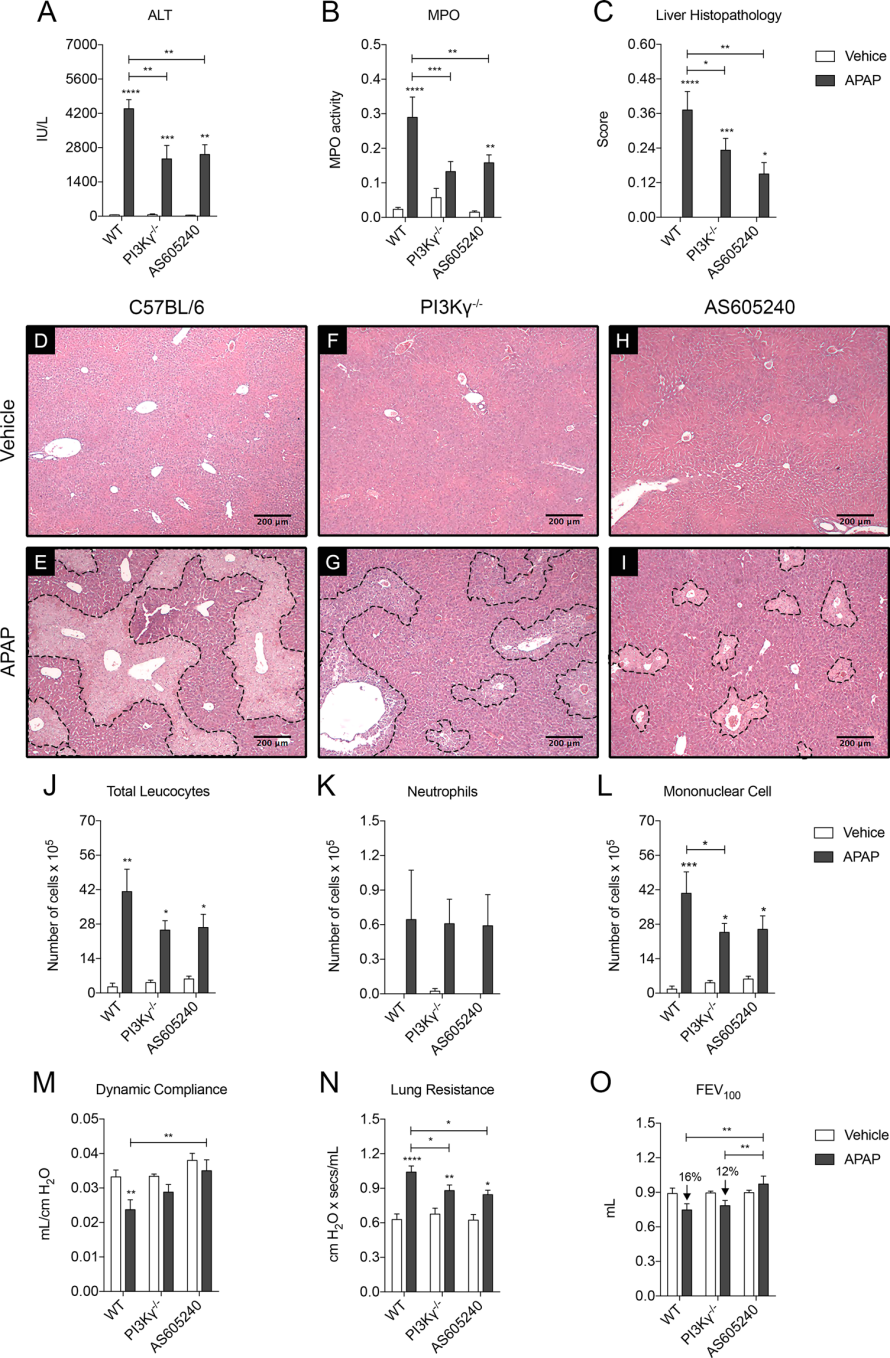
PI3Kγ deletion or inhibition prevents APAP-induced liver injury and lung remote damage. (**A**) ALT levels in the serum of WT (n = 5), PI3Kγ^−/−^ (n = 8) and AS605240 treated (n = 5) mice (20 mg/Kg). *p < 0.05; **p < 0.01; ****p < 0.0001. (**B**) MPO activity in the lung parenchyma of WT (n = 7), PI3Kγ^−/−^ (n = 12) and AS605240 treated (n = 7) mice (20 mg/Kg). *p < 0.05; **p < 0.01; ****p < 0.0001. (**C**) Histological score of livers from WT (n = 5), PI3Kγ^−/−^ (n = 7) and AS605240 treated (n = 5) mice (20 mg/Kg). *p < 0.05; ^**^p < 0.01; ****p < 0.0001. (**D** to **I**) Representative sections of liver lobes stained with Haematoxylin and Eosin; necrotic areas are delimited by dashed line. Bars 200 µm, magnification 40x. (**J** to **L**) Number of cells recovered in the BALF of WT (n = 5), PI3Kγ^−/−^ (n = 8) and AS605240 treated (n = 5) mice (20 mg/Kg) after APAP overdose (500 mg/Kg): total cells (**J**), neutrophils (**K**) and mononuclear cells **(L**). *p < 0.05; **p < 0.01; ***p < 0.001. (**M** to **O**) Lung function parameters of WT (n = 4), PI3Kγ^−/−^ (n = 8) and AS605240 treated (n = 4) mice (20 mg/Kg): Dynamic compliance (**M**), Lung Resistance (**N**) and Forced Expiratory volume at 100 ms (**O**). *p < 0.05 and ****p < 0.0001. Data represented as mean ± SEM. p-value was calculated using two-way ANOVA test with uncorrected Fisher’s LSD test.

We show that WT mice treated with an overdose of APAP had high levels of alanine transaminase (ALT), high liver MPO activity combined with large areas of necrotic tissue in the liver. However, when the APAP overdose was given to PI3Kγ^−/−^ mice or mice treated with AS6052540, the severity of the parameters associated with APAP-induced DILI was reduced. These mice had lower levels of ALT in the serum, lower MPO levels in the liver and less areas of necrotic tissue when compared to the WT mice (Fig. 4A–I). Quantification of the levels of total glutathione (GSH) in the liver and ALT in the serum of WT and PI3Kγ deficient mice and ALT in the serum of these animals revealed that both groups had the same amount of GSH and circulating ALT 2 hours after APAP administration (Supplementary Fig. 1). This indicates that these animals had no difference in APAP metabolism and in the onset of the disease, discarding the hypothesis that the differences observed in the APAP-induced liver injury was due to metabolic defects in these animals.

APAP overdose also caused remote lung damage in WT mice, as we previously described^10^. These mice had marked leukocyte infiltration to the airways predominantly composed of mononuclear cells and, to a lesser extent, neutrophils (Fig. 4J–L). This was accompanied by compromised lung function with reduced compliance, higher airway resistance, lower Forced Expiratory Volume (FEV_100_) (Fig. 4M–O) and histopathologic alterations, including leukocyte accumulation in the perivascular region and oedema (Supplementary Fig. 2). However, in the absence or inhibition of PI3Kγ, the remote lung damage caused by the APAP overdose was dampened. Fewer cells in the airways (due to the lower number of mononuclear cells recruited), higher lung compliance, lower airway resistance, higher FEV_100_, and lower histopathological score due to the reduced infiltration of leukocytes in the lung parenchyma and oedema were observed (Fig. 4J–O and Supplementary Fig. 2).

### Inhibition of PI3Kγ in both myeloid and parenchymal cell compartments is necessary for protection against APAP-induced liver injury

To increase our understanding of the mechanism by which PI3Kγ participates to TLR9-induced disease, we sought to determine in which tissue PI3Kγ was more relevant. To address this question, we generated chimeric mice by transferring bone marrow to WT or PI3Kγ^−/−^ mice from WT or PI3Kγ^−/−^ donor mice and performed the APAP-induced liver injury model.

**Figure 5:**
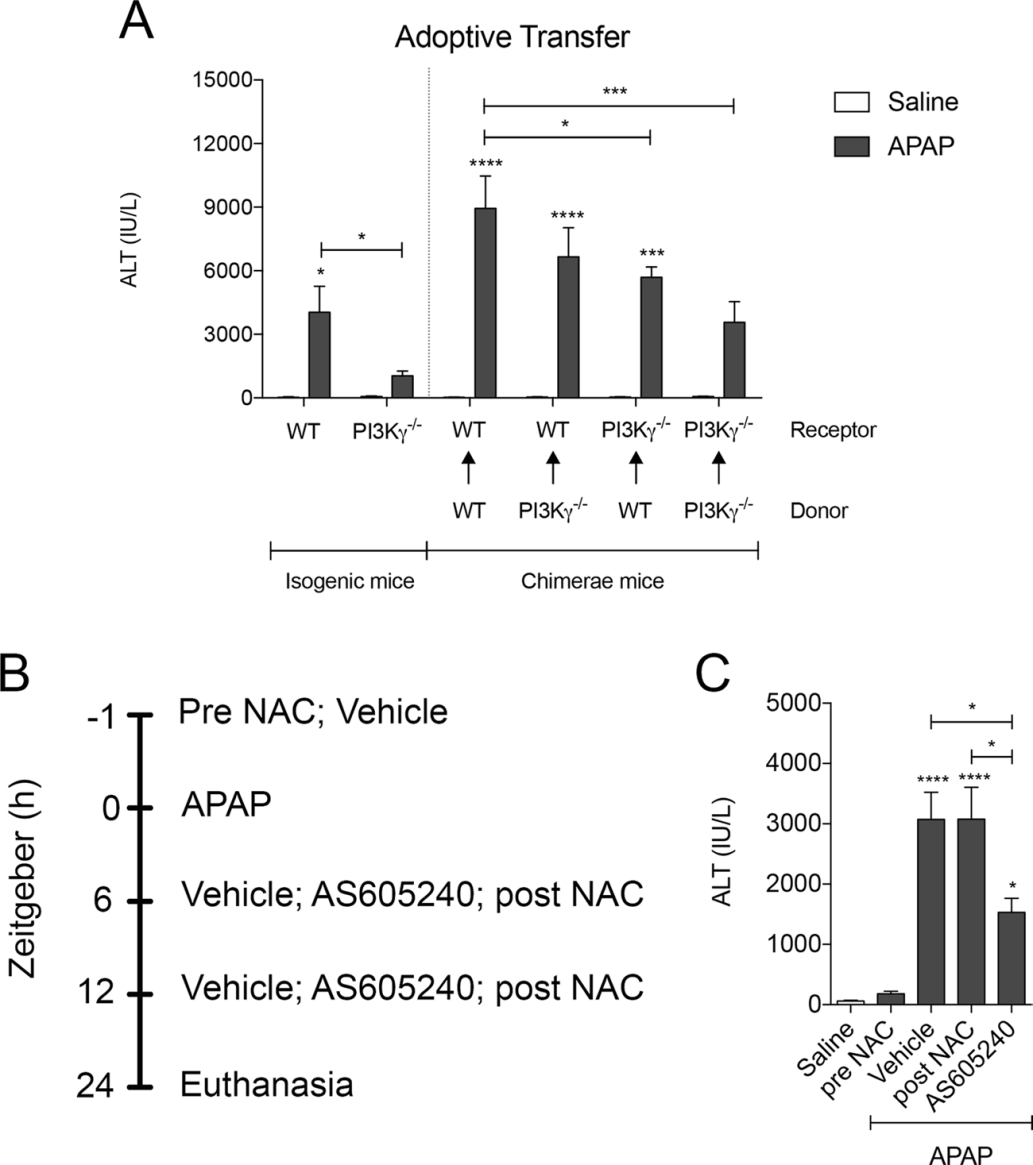
PI3Kγ inhibition in both parenchymal and myeloid tissue is a better treatment than POST NAC. (**A**) ALT levels in the serum of WT (n = 6), PI3Kγ^−/−^ (n = 7), WT/WT (n = 8), WT/PI3Kγ^−/−^ (n = 7), PI3Kγ^−/−^/WT (n = 8), PI3Kγ^−/−^/PI3Kγ^−/−^ (n = 5) after APAP overdose (500 mg/Kg). Data represented as mean ± SEM. p-value was calculated using two-way ANOVA test with uncorrected Fisher’s LSD test; *p < 0.05; **p < 0.01; ***p < 0.001. (**B**) treatment strategy to compare AS605240 (20 mg/Kg) and NAC (600 mg/Kg) treatments. (**C**) ALT levels in the serum of Vehicle (n = 5), APAP (n = 6), post NAC (n = 5), AS605240 (n = 4) and pre-NAC (n = 6) mice 24 h after APAP overdose. Data represented as mean ± SEM. p-value was calculated using one-way ANOVA test with uncorrected Fisher’s LSD test; ***p < 0.001; ****p < 0.0001.

Our results showed that APAP could induce liver damage in isogenic mice and that PI3Kγ^−/−^ mice were protected from disease. As expected, WT mice that received bone marrow from WT mice developed liver injury and PI3Kγ^−/−^ mice that received bone marrow from PI3Kγ^−/−^ mice were protected from it, as seen by ALT levels in the serum. Moreover, we found out that the group of WT mice that received the PI3Kγ^−/−^ bone marrow was not protected from the disease. However, analysing the group of PI3Kγ^−/−^ mice that received the WT bone marrow, we observed that there was in fact a partial protection from liver injury in this group, as shown by a mild reduction in serum ALT levels (Fig. 5A).

Finally, we compared the therapeutic benefit of the PI3Kγ inhibitor against N-acetyl-cysteine (NAC), the current standard of care for liver injury (Fig. 5B). As previously observed, APAP treatment induced high levels of serum ALT 24 hours after drug administration. As expected, prophylactic treatment with NAC completely abrogated the rise of ALT levels in the serum, whereas therapeutic treatment with NAC was not able to reduce ALT levels in the serum. In contrast, AS605240 treatment resulted in a reduction of approximately 50% in the serum ALT levels when given in a similar therapeutic schedule as NAC; ie. 6 and 12 hours after administration of APAP (Fig. 5C). This clearly demonstrate that timing is a crucial factor for DILI treatment and that PI3Kγ inhibition can broaden this window.

### PI3Kγ inhibition attenuates TLR9-induced cytokine production with no impact on cell viability

Our data suggested that PI3Kγ inhibition is a good target to treat sterile inflammation by reducing the inflammatory response and the outcomes of the studied diseases. However, several TLRs are expressed differently in mice and humans – differences include the transcription profile of TLRs in different cell types and regulation of cellular activation^42^. To address the issue of known differences in TLR expression and function between mice and humans, we switched to an *in vitro* CpG stimulated cytokine production in human PBMCs in the presence of the selective PI3Kγ inhibitor GSK723.

Before using the compound, the PI3Kγ selectivity of the GSK723 was tested. The inhibition of recombinant PI3Kα, PI3Kβ, PI3Kγ and PI3Kδ was measured using a homogeneous time-resolved fluorescence resonance energy transfer (TR-FRET) enzyme assay. A TR-FRET complex formed between a tagged GRP1 pleckstrin homology (PH) domain and a biotinylated PIP3 was used as a probe to quantify the PI3K catalysed formation of unlabelled PIP_3_ as previously described^43^. In this assay format, GSK723 gave average IC50 values of 15.8, 1.0, 0.0025 and 1.3 μM against PI3Kα, PI3Kβ, PI3Kγ and PI3Kδ, respectively. All other lipid or protein kinases tested using available in-house and external assays returned IC50 values greater than 10 μM including against close analogs such as PIK3C2B, PI4KA, PI4KB, Vps34, mTOR and DNA-PK (data not shown).

The cellular potency and selectivity of GSK723 was determined in basophil assays as previously reported^44^. Basophils can be activated by fMLP or anti-IgE via PI3Kγ or PI3Kδ dependent mechanisms, respectively, causing the basophil to degranulate and upregulate the tetraspanin CD63, a cell surface marker of basophil and mast cell activation. We profiled GSK723 in both fMLP and anti-IgE induced basophil surface CD63 expression as a means of confirming both the cellular potency of GSK723 for PI3Kγ and its selectivity against PI3Kδ. The IC50 values measured in this assay were 0.3 and 4.0 μM in the fMLP and anti-IgE assays, respectively (data not shown).

**Figure 6:**
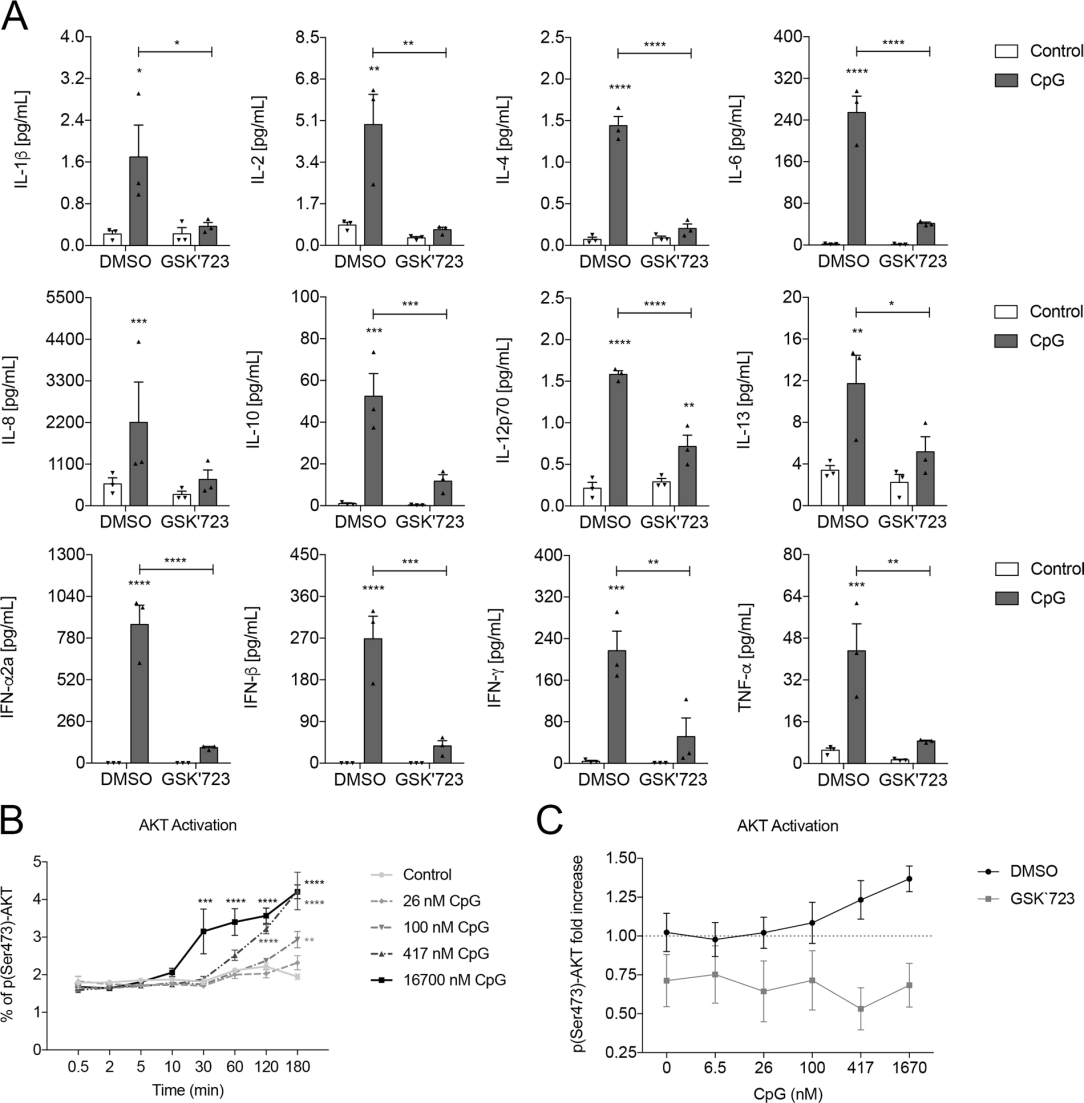
PI3Kγ is important for TLR9-induced cytokine production in PBMCs. (**A**) Cytokine production of PBMCs stimulated with CpG (100 nM) and the effect of GSK723 (3 µM) over cytokine production (n = 3 donors). Data represented as mean ± SEM. p-value was calculated using two-way ANOVA test with uncorrected Fisher’s LSD test; *p < 0.05; **p < 0.01; ***p < 0.001; ****p < 0.0001. (**B**) Activation of Akt by CpG in a time and concentration dependent manner (n = 3 donors). Data represented as mean ± SEM. p-value was calculated using one-way ANOVA test with uncorrected Fisher’s LSD test; *p < 0.05; **p < 0.01, ****p < 0.0001. (**C**) Fold change of pAkt levels over the basal level at 60 minutes after CpG stimulation (417 nM) of PBMCs with and without GSK’723 treatment (3 µM) (n = 3 donors). Control levels are represented by the dashed line and errors are 95% CI.

Once the selectivity of the compound has been confirmed, the effects of PI3Kγ inhibition on PBMCs stimulated with CpG was assessed. Stimulation of PBMCs in a concentration-response manner of CpG induced the production of all the cytokines measured, namely IL-1β, IL-2, IL-13, IL-8, IL-4, IFN-γ, IL-10, IL-6, TNF-α, IL12p70, IFN-β and IFN-α2a. Conversely upon PI3Kγ inhibition, the production of those cytokines was significantly dampened (Fig. 6A and Supplementary Fig. 3).

We also observed that upon CpG stimulation of PBMCs, there is an important rise in the levels of pAkt that is time and CpG concentration-dependent (Fig. 6B) and that, in presence of GSK723, the detection of pAkt is completely dampened (Fig. 6C). These results taken together indicated a relationship between PI3Kγ and TLR9 and translated our findings from pre-clinical models to a human system. We also investigated if the reduced cytokine production observed in the cells treated with GSK723 were due to cytotoxic effects. Our results showed that no change in cellular viability was observed after treatment with GSK723 and stimulation with CpG (Supplementary Fig. 4).

## Discussion

Although TLRs and PI3Ks are very important proteins for the host immune response, both were only discovered relatively recently^5,45^. Their discoveries spurred studies towards the elucidation of how they may interact to modulate the immune response^31,46,47^. Interestingly, these studies have shown different functions or even conflicting roles for PI3Ks depending on the context of the study. The lack of consistencies may have been caused in part by the use of non-selective PI3K inhibitors, especially Wortmannin and LY294002^48^ and the fact that these enzymes were widely studied in non-physiologically relevant contexts, such as transfected cell lines, which did not replicate the correct signalling events^49,50^. In addition, the systems used were monoculture of cells such as THP-1, pDCs, human tracheal smooth muscle cells (HTSMCs), mouse macrophages and colonic epithelial cells^28,33–36,51^.

Here we sought to examine the relationship of TLR9 and PI3Kγ *in vivo*. We adapted an LPS-induced pleurisy model previously reported by our group^52^ to that of a CpG-induced pleurisy so we could demonstrate the role of PI3Kγ in an *in vivo* system with multiple cells in play. We found that CpG induced an immune response similar to that previously observed with LPS, with an acute peak of neutrophil recruitment followed by the influx of mononuclear cells. We also showed that PI3Kγ is critical for the recruitment of cells in the pleura because when absent or inhibited, there were fewer cells detected in the pleura. However, we could not rule out that the PI3Kγ deletion or inhibition had an indirect effect on those cells migrating rather than blocking TLR9. We thus further evaluated the effect of PI3Kγ inhibition in alternative TLR9-dependent models with additional endpoints other than cell recruitment.

The model of airway silicosis in which an irreversible lung injury is caused by the inhalation of silica particles was first chosen^53^. Although there was no published direct evidence of TLR9 involvement in silicosis, other authors have demonstrated a correlation between TLR9 and pulmonary fibrotic diseases such as idiopathic pulmonary fibrosis or fibrosis induced by paraquat^54–56^. In such a model, the activation of the NLRP3 inflammasome leads to the release of pro-inflammatory and pro-fibrogenic cytokines such as IL-1β, TNF-α, IL-6, and TGF-β^57,58^. Here, we clearly demonstrate that, in the absence of TLR9, these animals were protected from silicosis since they showed less lung inflammation, reduced fibrosis and improved lung function. In addition, exploring the role of PI3Kγ in the context of pulmonary fibrosis, our group demonstrated that PI3Kγ^−/−^ mice are protected from lethality, lung inflammation and fibrosis when challenged with bleomycin *in vivo*^59^. Taking this into account, we further strengthened the relevance of PI3Kγ in TLR9-dependent inflammation by showing that the absence of PI3Kγ is beneficial to the host exposed to silica thereby recapitulating the outcome also observed with the TLR9^−/−^ mice. Of note, to the best of our knowledge, we are the first group to demonstrate a correlation between the TLR9 and PI3Kγ pathways for the development of silicosis.

Our second model of choice was the APAP-induced liver injury. APAP is the most used analgesic and antipyretic drug for the treatment of mild inflammatory symptoms. Although it is safe under therapeutic doses, overdose of APAP may lead to acute liver injury and mortality^60,61^. Previous studies from our group have shown that APAP intoxicated livers have large areas with DNA deposition co-localising with necrotic areas. Furthermore, we demonstrated that TLR9^−/−^ mice have less paracetamol-induced liver necrosis, ALT and TNF-α levels in the serum^41^. APAP-induced liver injury is a metabolism dependent disease, thus changes in the metabolism rate may affect the course and severity of the disease since different levels of the toxic NAPQI byproduct might be generated. Considering that PI3Kγ is also involved in cellular metabolism, blocking this enzyme might naturally affect the course of the disease. We ruled out this hypothesis by demonstrating that, after 2 hours of APAP administration, both WT and PI3Kγ^−/−^ animals have the same levels of reduced GSH in the liver, indicating that the metabolic rate of both animals is the same. Moreover, we also show that both animals have the same amount of circulating ALT at the onset of the disease. Taken together, these data suggest that the differences observed between WT and PI3Kγ^−/−^ animals might be due to a process occurring during the disease development and not in its genesis.

PI3Kγ is preferentially expressed in haematopoietically derived cells and is known to play critical roles in leukocyte biology^14,20,45^. The functions of PI3Kγ in inflammatory diseases have hence been reported through the use of selective tool inhibitors with respect to reducing leukocyte-derived migration^17^, activation^59,62^, cytokine production^33^, angiogenesis and fibrosis^59^. However, the expression of this enzyme in cells other than leukocytes has also been proposed, for example in hepatocytes or lung epithelial cells^63,64^. To understand the role of the PI3Kγ in non-hematopoietic cells, we evaluated the response of chimera mice to the paracetamol-induced liver injury. Interestingly we found out that the mice were protected when PI3Kγ was absent in both the hematopoietic and parenchymal cells, however, we also found that animals with a PI3Kγ^−/−^ background that received bone marrow from WT mice also had some level of protection. This finding highlights an uncovered but important role for PI3Kγ in an unexpected tissue, given that its expression is expected to be mostly restricted to leukocytes.

Although DILI and silicosis are very different models, they share common features. Despite the predominant expression in leukocytes, it has been suggested that other cellular types such as hepatocytes and lung epithelial cells also express PI3Kγ and TLR9^63–66^. Also, according to the literature and the data showed in this study, excessive paracetamol and exposure to silica leads to mitocondrial damage, release of DNA and mitophagy/autophagy^67–69^. As a consequence, the mitochondrial DNA (mitDNA) released during these processes might well be a mechanism by which TLR9 could be activated and could in turn activate PI3Kγ as suggested by our data. Interestingly, the activation of the Akt/mTOR axis, has also been reported to block the cellular autophagy^70,71^. The dysregulation of the mitochondrial turnover through the interference of mitogenesis or mitophagy may cause an imbalance in the energy produced in these cells which could cause cell death. Thus, in this context, PI3Kγ could have a critical role in the onset of the disease. This proposed mechanism has recently been reported by our group, however in a different model of sterile inflammation involving cardiomyocyte damage induced by doxorubicin^72^.

After liver injury is diagnosed, the treatment options available include removal of the causing agent, administration of the standard of care NAC or liver transplant^73,74^. The time window for NAC administration is very short and the patients must be treated in the initial phase of the disease, within the first 12–24 hours^75^. Because of the short time window and low efficacy of NAC, alternative treatments are sought after. In our study, treatment with AS650240 was also effective post APAP challenge with 50% reduction in blood ALT levels whilst NAC had no impact in that setting. This suggests that inhibition of the PI3Kγ could offer therapeutic options for the treatment of acute liver injury that are superior to NAC.

Our study demonstrates that PI3Kγ inhibition resulted in relevant outcomes for serious diseases such as silicosis and DILI. Given the importance of these results we decided to investigate whether these findings could be translated to humans. Our experiments revealed that, upon stimulation with CpG, Akt is phosphorylated and that treatment with GSK723 reduced both basal and stimulated pAkt. These results establish a link between TLR9 and PI3Kγ activation. Interestingly, the levels of pAkt start to rise between 10 to 30 minutes after CpG stimulation suggesting that it is most likely resulting from indirect stimulation since direct PI3K activation typically results in Akt phosphorylation within five minutes of stimulation^76^. Overall, our results indicate a clear interplay between TLR9 and PI3Kγ, however, future studies are required to address mechanism leading to PI3Kγ activation downstream of TLR9 signalling whether direct or due to GPCR mediated paracrine/autocrine activation loops.

In conclusion, the present study demonstrates that PI3Kγ has a critical role in some immune responses mediated by TLR9, which is supported by our findings using *in vitro* and *in vivo* CpG-induced models. Whilst a functional link is provided by the CpG models, we cannot unequivocally establish that TLR9 and PI3Kγ activation are dependent of each other in the more complex models of APAP injury or silicosis due to the limitations of the experimental approach. For example, inflammatory mediators such as chemokines or cytokines rather than endogenous TLR9 ligands could also activate PI3Kγ^77^. Nevertheless, our study reports that there is a critical role for PI3Kγ in the TLR9-dependent inflammatory models we tested. This suggests that regardless of the possible involvement of other pathways in these more complex models, the inhibition of PI3Kγ has a significant impact on TLR9-induced inflammatory responses. Altogether, this work provides information that warrants further investigation of PI3Kγ as a therapeutic option for the treatment of disorders where excessive TLR9 activity is implicated.

## Material and Methods

### Pleurisy model

Animals received one single injection of C-class CpG in the pleural cavity with the indicated dose of agonist. At indicated timepoints, mice were anesthetized intraperitoneally with a mixture of Ketamine/Xylazine and then killed with overdose of intravenous anaesthetic (80 mg/Kg and 15 mg/Kg, respectively). Pleural cavity was washed two times with 1 mL of PBS 10 mM EDTA resulting in a total of 2 mL of lavage. Cells were centrifuged and used for total cell count and differential cell count after Romanowsky staining (Panótico Rápido LB). For the pleurisy model, mice were orally treated with AS605240 twice: 1 h prior the CpG injection and then 6 h after CpG, 20 mg/kg diluted in 0,9% Saline + 0.5% CMC + 0.1% Tween 20.

### Drug induced liver injury induction

Mice were fasted for 15 hours before oral APAP administration (500 mg/Kg) or vehicle (sterile saline). After 24 hours mice were anesthetized for respiratory mechanics analysis (described below) and then killed for blood (serum), liver, bronchoalveolar lavage fluid (BALF), and lung collection^10^. Alanine aminotransferase (ALT) levels were measured in the serum using an enzyme activity test (Bioclin). The myeloperoxidase activity assay was performed as described before^78^. BALF was performed by washing the lungs twice with 1 mL of PBS 10 mM EDTA with a polyethylene cannula 18 G resulting in a total of 2 mL of lavage. Cells were centrifuged and used for total cell count and differential cell count after Romanowsky staining (Panótico Rápido LB)^59^. Fragments of liver and the left lobe of the lungs were fixed and then sectioned for histological analysis (Haematoxylin and Eosin). For the drug-induced liver injury model, mice were orally treated with AS605240 6 and 12 hours after APAP treatment or with n-Acetylcysteine (NAC) 1 hour before APAP or 6 and 12 hours after APAP treatment. AS605240 was diluted in 0,9% Saline + 0.5% CMC + 0.1% Tween 20 and given in a dose of 20 mg/Kg and NAC was diluted in 0.9% saline and given in a dose of 600 mg/Kg.

### Silicosis induction

Mice were anesthetized with a mixture of Ketamine/Xylazine and then instilled with 40 µL of sterile saline containing 10 mg of silica (Sigma) or saline alone^53^. Sample collection and lung function were accessed 28 days after silica instillation. Lung mechanics was assessed (described below) and BALF was collected as mentioned before and total and differential cell count were performed. The presence of macrophage in the lung parenchyma was assessed by β-N-Acetylglucosaminidase (NAG) Activity Assay and normalised to the number of macrophages harvested from the peritoneal cavity of mice injected with 5% thioglycolate^78^. The left lobe of the lung was fixed and then sectioned for histological analysis for inflammation (Haematoxylin and Eosin) and fibrosis (Gomori Trichrome). The amount of cell free DNA (cfDNA) measured in the BALF was performed using Spectrophotometer ND-1000 (NanoDrop).

### Invasive analysis of the respiratory mechanics

Animals were anesthetized with a mixture of Ketamine/Xylazine (200 mg/Kg and 15 mg/Kg, respectively) as indicated by Buxco. Dynamic compliance (mL/cm H_2_O), lung resistance (cm H_2_O × s/mL) and Forced Expiratory Volume at 100 ms (FEV_100_) (mL) were assessed in tracheostomized and mechanically ventilated mice using Forced Manoeuvres Platform (Buxco)^53^.

### Chimera mice

To generate chimera mice, WT and PI3Kγ^−/−^ had two sessions of gamma irradiation (Cobalt 60). In the first round, mice were submitted to 4 Gy of absorbed radiation and, three hours later, to 5 Gy. Immediately after the second irradiation the mice received 1 × 10^7^ cells derived from the bone marrow (no RBCs) intravenously. The mice generated were: WT → WT; WT → PI3Kγ^−/−^; PI3Kγ^−/−^ → WT; PI3Kγ^−/−^ → PI3Kγ^−/−^. Antibiotics were given to these mice from one day prior to the radiation until 10 days after the procedure. Six weeks after the adoptive transfer total circulating cells were counted to confirm if the procedure were successful^79^.

### PBMC isolation, stimulation and treatment

PBMC cells were prepared from heparinised human blood (using 1% v/v Heparin Sodium 1,000 IU/ml Endotoxin Free, Leo Laboratories) from normal volunteers with the Accuspin System and Histopaque-1077 (Sigma-Aldrich) and gradient centrifugation at 400 g for 35 minutes at 25 °C. Cells were resuspended in RPMI 1640 supplemented with 1.5 mM L-Glutamine and 10% FBS (all from Gibco) and seeded into 96-well round bottom cell culture plate (Nunc) at a density of 10^6^ cells per well in presence or absence of 3 µM GSK723. After an hour incubation, the cells were stimulated with different concentrations of C-class CpG for 24 hours otherwise stated.

### Cytokines and Akt phosphorylation measurements

Cytokines (IFN-α2a; IFN-β; IFN-γ; IL-1β; IL-2; IL-4; IL-6; IL-8; IL-10; IL-12p70; IL-13; TNF-α were measured) 24 hours from the PBMCs supernatant using the MSD platform according to the manufacturer’s instruction (Meso Scale Diagnostics).

Akt phosphorylation was also measured using the MSD platform. At various indicated timepoints, the cells were lysed on ice with lysis buffer containing anti-proteases and anti-phosphatases provided in the MSD kit. The amount of total and phosphorylated Akt were measured following the manufacturers instruction and the results were represented as percentage of phosphorylated protein or as fold increase using the equation $\frac{1}{\log (\frac{CPD}{DMSO})}$.

### Statistics

Statistical analysis was performed in Prism version 7.0 (www.graphpad.com). Before statistical analysis, the datasets were analyzed regarding its normality using the D’Agostino-Pearson omnibus normality test or Kolmogorov-Smirnov test. Once the normality of the data was stablished, were used: non-parametric Kruskal-Wallis test followed by uncorrected Dunn’s test, one-way ANOVA test followed by uncorrected Fisher’s LSD test or two-way ANOVA followed by uncorrected Fisher’s LSD test as stated in the figure legends. Differences were considered significant at *p ≤ 0.05, **p ≤ 0.01, ***p ≤ 0.001 and ****p ≤ 0.0001.

### Study approval

Peripheral blood was collected from healthy volunteers by Venepuncture with prior approval from the East of England Cambridgeshire and Hertfordshire Research Ethics Committee. All volunteers provided written consent prior to donation and after being informed of the end use of their blood sample. All procedures were carried out in strict accordance to their informed consent. Donor identification was coded to preserve donor confidentiality. The PI3Kγ selective inhibitor GSK723 was synthesised as previously described^80^. All the procedures were approved by the NRES Committee East of England – Cambs & Herts Reserach Ethics Committee, No. 07/H0311/103 and the procedures were performed in accordance with the relevant guidelines and regulations.

In this study, female C57BL/6 mice from 8 to 12 weeks of age were used in all experiments except for the chimaera mice. Animals were obtained from the UFMG’s Centro de Bioterismo and kept in the Laboratório de Imunofarmacologia with food and water *ad libitum* and day/night light cycles. For all investigations, mice were humanely sacrificed using an intra-peritoneal overdose of Ketamine/Xylazine at a dose equivalent to at least five times the dose used for anesthetising the mice. The animal studies presented here were registered and approved by the UFMG’s Comitê de Ética em Utilização Animal (CEUA) No. 191/2010 and 51/2011 and all the procedures were performed in accordance with the guidelines of the Conselho Nacional De Controle De Experimentação Animal’s resolution of Brazilian Ministry of Science, Technology, Innovation and Communication.

### Summary

Lima *et al*. demonstrate that PI3Kγ is necessary for TLR9-dependent cellular activation. The presence of PI3Kγ is shown to be as important in leukocytes as in non-hematopoietic cells for the development of diseases that are caused/exacerbated by the recognition of DNA via TLR9.

## Supplementary information

Supplemental data

## Data Availability

All the data is available within the maniscript.
