# Supplementary material for: Converging TLR9 and PI3Kgamma signaling induces sterile inflammation and organ damage
Source: Sci Rep. 2019 Dec 13;9:19085. doi: 10.1038/s41598-019-55504-0 (PMC6910931; doi:10.1038/s41598-019-55504-0)
Supplement: Supplementary file 1 — Supplemental data [file 41598_2019_55504_MOESM1_ESM.pdf]

Converging TLR9 and PI3Kgamma signaling induces sterile inflammation and organ damage

Braulio Henrique Freire Lima[1]; Pedro Elias Marques[1]; Lindisley Ferreira Gomides[2]; Matheus Silvério Mattos[3]; Lucas Kraemer[3]; Celso M. Queiroz-Junior[4]; Mark Lennon[5]; Emilio Hirsch[6]; Remo Castro Russo[3]; Gustavo Batista Menezes[2]; Edith M. Hessel[7]; Augustin Amour[7†]; Mauro Martins Teixeira[1†*]

[1]Department of Biochemistry and Immunology, Institute of Biological Sciences, Feredal University of Minas Gerais, Belo Horizonte, Minas Gerais, Brazil;

[2]Center for Gastrointestinal Biology, Instituto de Ciências Biológicas, Feredal University of Minas Gerais, Belo Horizonte, Minas Gerais, Brazil;

[3]Physiology and Biophysics/Instituto de Ciencias Biologicas, Feredal University of Minas Gerais, Belo Horizonte, Minas Gerais, Brazil;

[4]Departament of Morphology, Institute of Biological Sciences, Feredal University of Minas Gerais, Belo Horizonte, Minas Gerais, Brazil;

[5]Target Sciences, GlaxoSmithKline, Stevenage, Hertfordshire, Stevenage, United Kingdom;

[6]Department ot Molecular Biotechnology and Health Sciences, Molecular Biotechnology Center, University of Torino, Torino, Italy

[7]Refractory Respiratory Inflammation DPU, GlaxoSmithKline, Hertfordshire, Stevenage, United Kingdom;

†Authors have contributed equally for the paper

Running Head: PI3Kγ and TLR9 converge in models of inflammation

Key words: PI3Kg, silica, inflammation, silicosis, DILI, APAP

Corresponding author: Mauro Martins Teixeira (mmtex.ufmg@gmail.com)

Supplementary Material and Methods

Total liver GSH measurement

Total liver GSH was measured following already described[1]. Briefly, 150 mg of liver tissue was homogenized in 150 µL of PBS. After homogenization, 300 µL of trichloroacetic acid (TCA) was added and tissue was homogenized again. Then the samples were centrifuged at 847 g at 4ºC for 15 minutes. Supernatant was collected for assay and pellet discarded. Fourty µL of blank and samples were pipetted in a 96-well plate in duplicates. After, 240 µL of Tris-HCl 0.4 M and 10% TCA were added to the samples. At last, 20 µL of DNTB 2,69 mg/mL in methanol were added to the samples. The plate was read in a spectrophotometer at 415 nm 30 seconds after the addition of DNTB. The assay was normalized by a 2-fold GSH standard curve starting at 500 µg/mL.

Heatmap generation

Heatmap was generated using the free software Multiexperiment Viewer v4.8.1 program that is available at http://www.tm4.org/mev.html. The results obtained from the MSD and ELISA assays were log_{10} transformed and plotted as a matrix where the cytokines were in the y axis and donors, treatments, and agonist concentration in the x axis. Zero values were considered 10-fold lower than the lowest value in the correspondent group. Minimum and maximum saturation values were defined as -1.0 and 3.5, respectively. Hierarchical clustering of the cytokines was performed uisng Pearson's correlation.

Cell viability assay

For the PBMCs viability assay, 10^5 cells/well were seeded into a 96-well round bottom cell culture plate, treated with 1 μM of GSK'723 for 1 hour and then stimulated with 0.4175 μM of C-class CpG. 24 hours later, the cells were washed and labelled with Annexin V-PI with BD Pharmigen™ FITC Annexin V Apoptosis Detection Kit I (Becton Dickinson) and ran into BD Accuri Cytometer (Becton Dickinson). Data was analysed using FloJo software.

Supplementary Figures

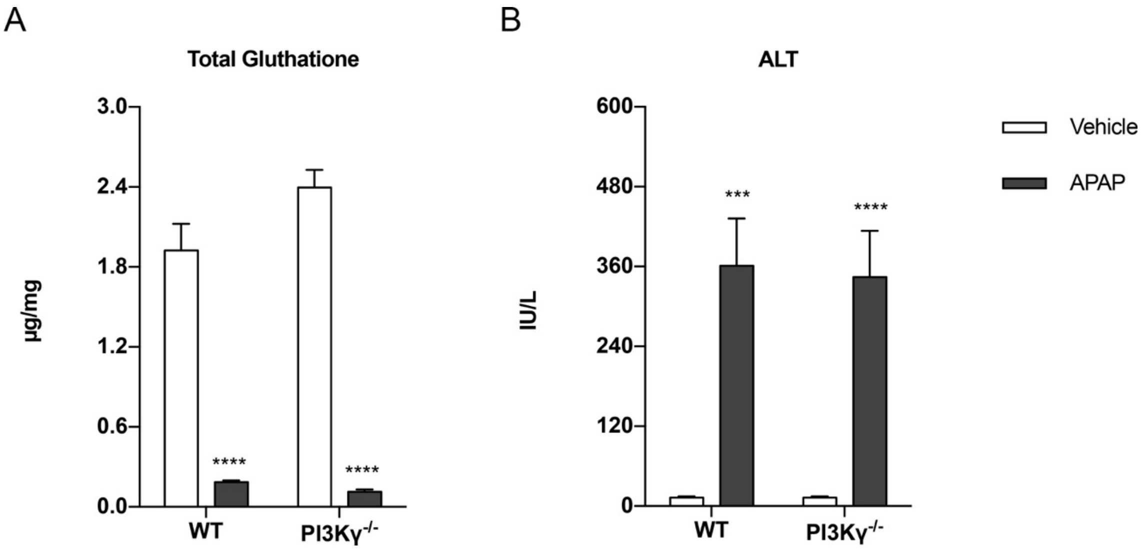

Supplementary Fig 1. WT and PI3Kγ$^{-/-}$ animals have similar levels of total liver GSH and serum ALT in the onset of DILI. (A) Levels of total GSH in the liver of WT (n=5) and PI3Kγ$^{-/-}$ (n=5). (B) ALT levels in the serum of WT (n=5), PI3Kγ$^{-/-}$ (n=5). Samples were analysed 2 hours after APAP administration (500 mg/Kg). Data represented as mean ± SEM. p-value was calculated using two-way ANOVA with uncorrected Fisher's LSD test; ***, $p < 0.001$; ****, $p < 0.0001$.

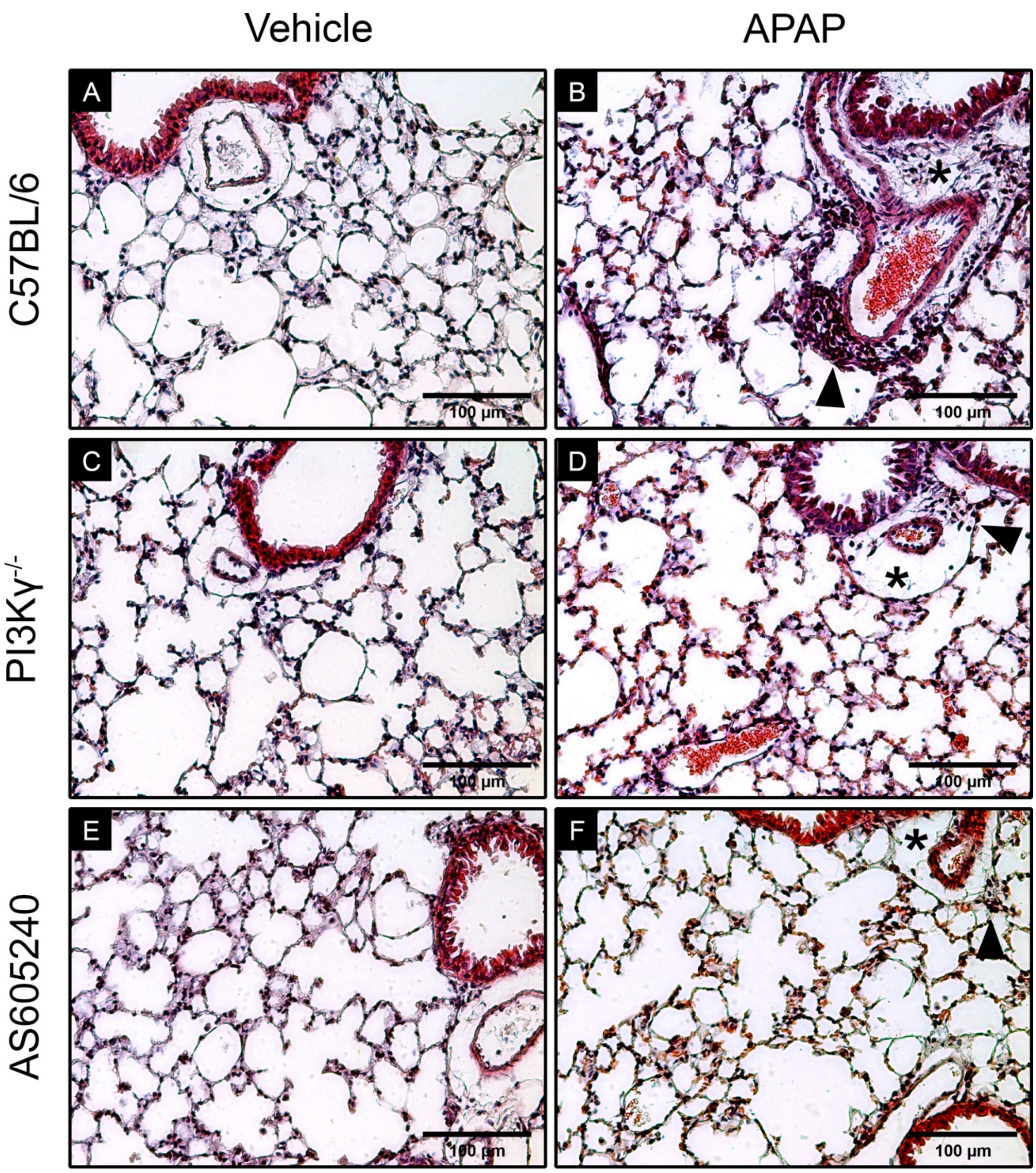

Vehicle
APAP
C57BL/6
PI3Kγ-/-
AS605240
A
B
C
D
E
F
100 µm

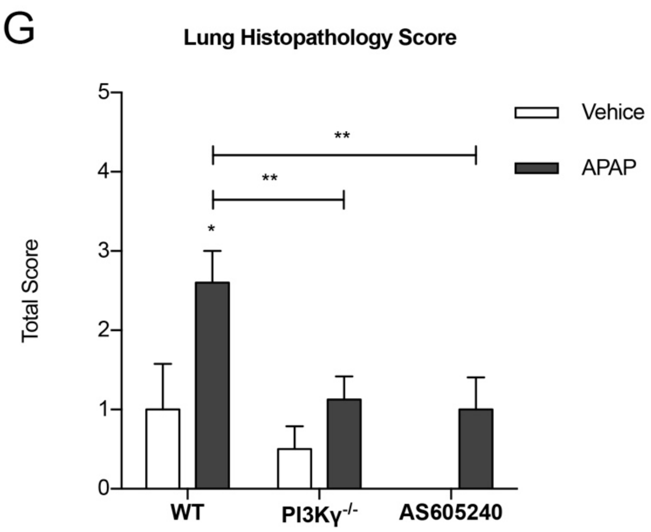

G
Lung Histopathology Score
Total Score
Vehice
APAP
WT
PI3Kγ-/-
AS605240
**
**
*

Supplementary Fig. 2. PI3Kγ deletion or inhibition reduce lung remote damage. (A) Representative sections of lung lobes stained with Haematoxylin and Eosin. Arrowheads indicate leukocyte infiltration and asterisks indicate perivascular oedema. Bars 100 µm, magnification 200x. (B) Histological score of the lung slides of WT (n=5), PI3Kγ$^{-/-}$ (n=8) and AS605240 treated (n=5) mice (20 mg/Kg) after APAP (500 mg/Kg) overdose. Data represented as mean ± SEM. p-value was calculated using two-way ANOVA with uncorrected Fisher's LSD test; *, $p < 0.05$; **, $p < 0.01$.

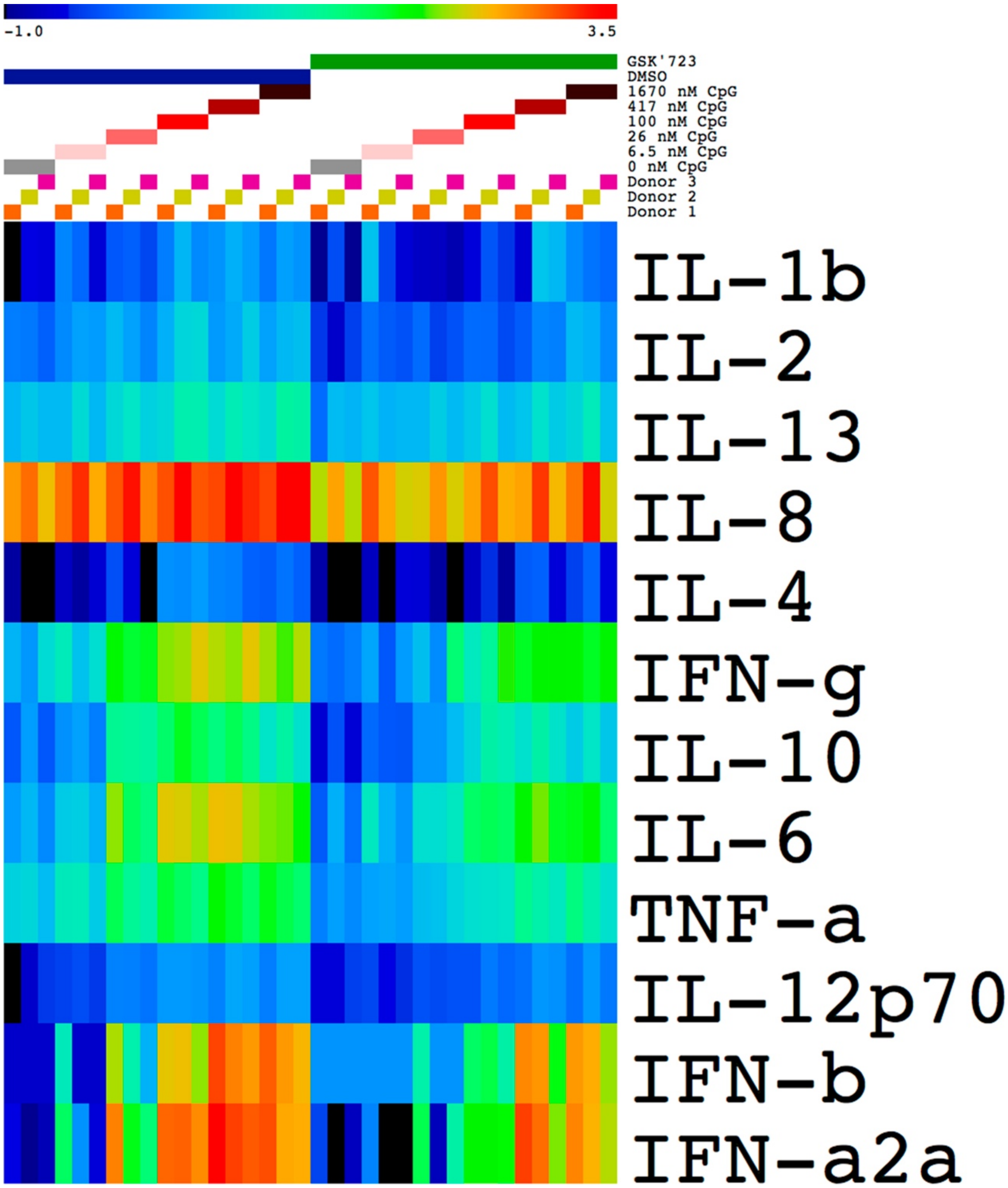

Supplementary Fig. 3. PI3Kγ inhibition dampen cytokine production of human PBMCs stimulated with CpG. Heatmap representing the concentration-response curve of CpG and the effect of GSK'723 (3 µM) on cytokine production. Data is expressed as $\log_{10}$ of the ELISA readout. Not detected values were expressed as 10-fold lower than the lowest value in the group.

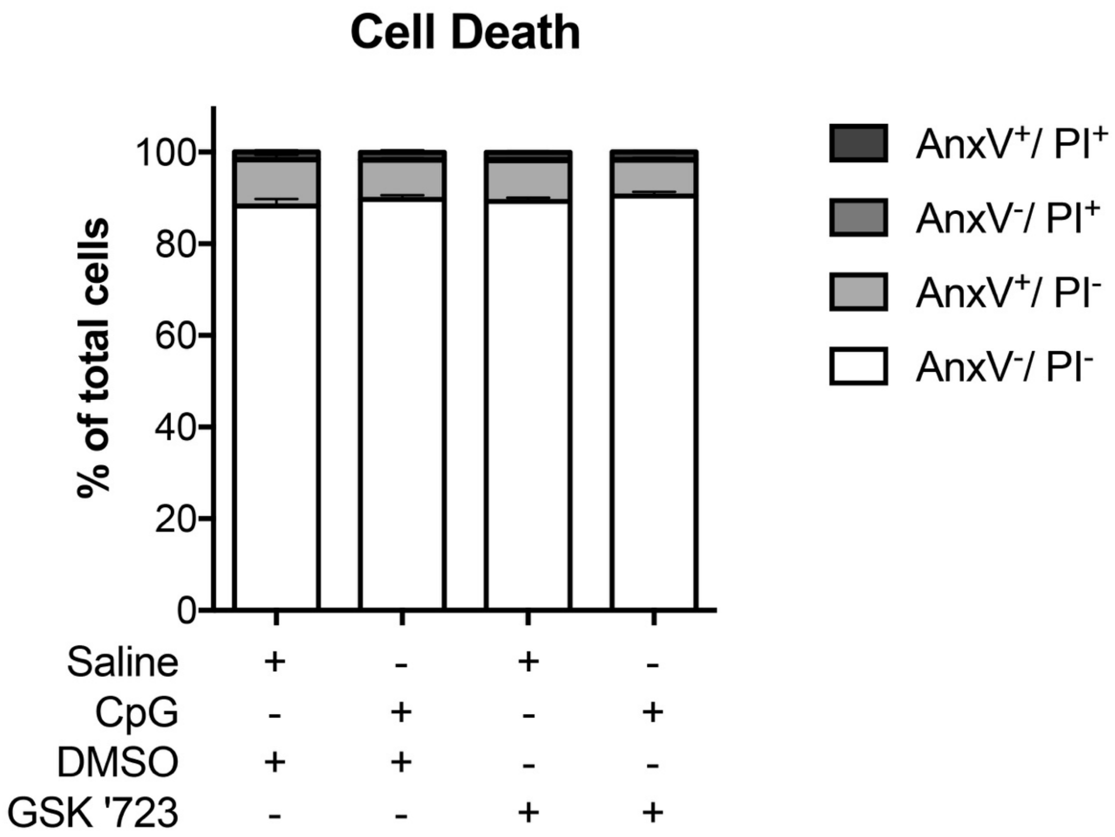

Supplementary Fig. 4. Treatment of CpG-stimulated human PBMCs with GSK'723 do not reduce its viability. PBMCs viability 24 hours after treatment with GSK'723 (3 µM) and stimulation with CpG (417 nM) (n=3 donors). Data represented as mean ± SEM. p-value was calculated using one-way ANOVA with uncorrected Fisher's LSD test.

References

1 Rahman, I., Kode, A. & Biswas, S. K. Assay for quantitative determination of glutathione and glutathione disulfide levels using enzymatic recycling method. *Nature protocols*, doi:papers2://publication/doi/10.1038/nprot.2006.378 (2006).
